# Are Redox‐Active Organic Small Molecules Applicable for High‐Voltage (>4 V) Lithium‐Ion Battery Cathodes?

**DOI:** 10.1002/advs.202200187

**Published:** 2022-03-10

**Authors:** Yuto Katsuyama, Hiroaki Kobayashi, Kazuyuki Iwase, Yoshiyuki Gambe, Itaru Honma

**Affiliations:** ^1^ Institute of Multidisciplinary Research for Advanced Materials Tohoku University 2‐1‐1 Katahira, Aoba‐ku Sendai Miyagi 980–8577 Japan; ^2^ Department of Chemistry and Biochemistry University of California, Los Angeles Los Angeles CA 90095 USA

**Keywords:** high energy densities, croconic acid, high voltage cathode materials, multi‐electron redox reactions, organic lithium‐ion batteries

## Abstract

While organic batteries have attracted great attention due to their high theoretical capacities, high‐voltage organic active materials (> 4 V vs Li/Li^+^) remain unexplored. Here, density functional theory calculations are combined with cyclic voltammetry measurements to investigate the electrochemistry of croconic acid (CA) for use as a lithium‐ion battery cathode material in both dimethyl sulfoxide and *γ*‐butyrolactone (GBL) electrolytes. DFT calculations demonstrate that CA dilitium salt (CA–Li_2_) has two enolate groups that undergo redox reactions above 4.0 V and a material‐level theoretical energy density of 1949 Wh kg^–1^ for storing four lithium ions in GBL—exceeding the value of both conventional inorganic and known organic cathode materials. Cyclic‐voltammetry measurements reveal a highly reversible redox reaction by the enolate group at ≈4 V in both electrolytes. Battery‐performance tests of CA as lithium‐ion battery cathode in GBL show two discharge voltage plateaus at 3.9 and 3.1 V, and a discharge capacity of 102.2 mAh g^–1^ with no capacity loss after five cycles. With the higher discharge voltages compared to the known, state‐of‐the‐art organic small molecules, CA promises to be a prime cathode‐material candidate for future high‐energy‐density lithium‐ion organic batteries.

## Graphical abstract

Croconic acid (CA) is investigated as a high‐energy‐density organic cathode material for lithium‐ion batteries. Its theoretical energy density is beyond those of most conventional materials thanks to its high‐voltage (>4 V) redox potentials of the two enolate groups. The electrochemical experiments confirm that CA undergoes reversible multi‐electron redox reactions around 4 V, which can be utilized for energy storage.

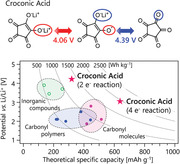

## 1 Introduction

The world's economic development and its increasing energy consumption require the shift of electricity generation from fossil fuels to renewable energy sources.^[^ ^1^, ^2^, ^3^ ^]^ To this end, the dependence of renewable energies on climate variability demands evermore performant energy storage devices to compensate for the unpredictable productivities.^[^ ^4^, ^5^, ^6^, ^7^ ^]^ Although the production of conventional inorganic secondary batteries has made great strides, their high cost and limited material resources remain challenges to meeting the needs of large‐scale renewable energy storage.^[^ ^8^, ^9^, ^10^, ^11^ ^]^

Recently, organic batteries—which are composed of ubiquitous elements, such as carbon, hydrogen, oxygen, and nitrogen—have gained much attention because they are inexpensive and can be synthesized semi‐permanently.^[^ ^9^, ^12^, ^13^, ^14^, ^15^, ^16^ ^]^ Further, organic active materials can be prepared from renewable resources such as biomass, contributing to the reduction of both energy consumption and CO_2_ emissions during mass production.^[^ ^9^, ^11^, ^12^, ^13^, ^15^, ^16^, ^17^, ^18^, ^19^ ^]^ In addition, the properties of organic molecules, such as redox potential, solubility, and capacity, are tunable by molecular engineering.^[^ ^12^, ^15^, ^20^ ^]^

One strategy for improving the energy density of lithium‐ion organic batteries is to design cathode active materials that can undergo multi‐electron redox reactions at higher voltages. However, to the best of our knowledge, only a few organic active materials that undergo multi‐electron redox reactions at voltages around 4 V against Li/Li^+^ has been reported to date,^[^ ^21^, ^22^ ^]^ whose theoretical capacities (*C*_th_) are relatively small (≈250 mAh g^–1^) since they have large molecular weights and the limited number of redox centers. Examples for high‐capacity organic cathode materials including the *π*‐conjugated quinoxaline‐based heteroaromatic molecules (≈2.9 V, C_th_ = ≈514 mAh g^–1^),^[^ ^23^, ^24^ ^]^ carbonyl‐based organic polymers (≈2.5 V, C_th_ = ≈440 mAh g^–1^),^[^ ^25^, ^26^, ^27^, ^28^, ^29^ ^]^ tetraaminoanthraquinone (≈3.0 V, C_th_ = ≈400 mAh^–1^),^[^ ^30^ ^]^ N,N’–substituted phenazine (≈3.7 V, C_th_ = ≈255 mAh g^–1^),^[^ ^21^ ^]^ and dibenzo‐1,4‐dioxin–tetracyanoquinodimethane (≈4.2 V, C_th_ = ≈200 mAh g^–1^),^[^ ^22^ ^]^ none of them can simultaneously achieve a high‐voltage discharge > 4 V against Li/Li^+^ and a high capacity > 400 mAh g^–1^.

**Figure 1:**
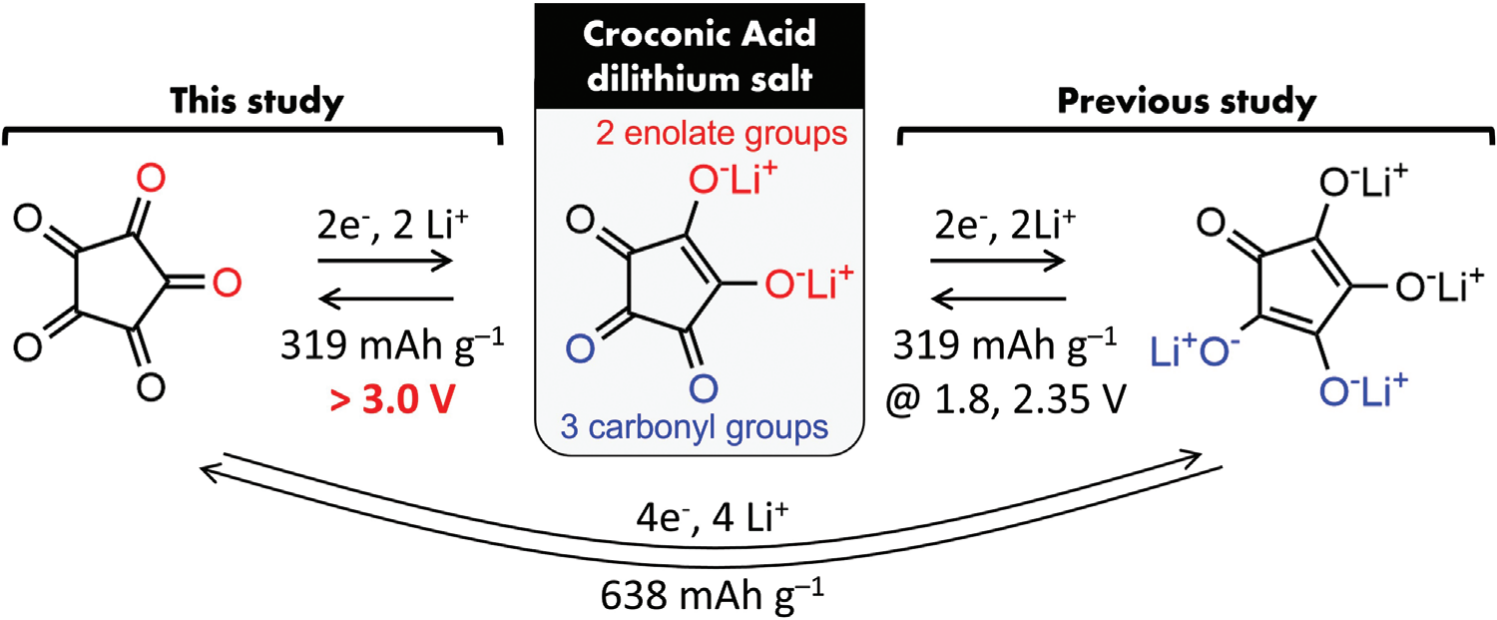
Conceptual illustration of this work on croconic acid with multi‐electron redox reaction at high voltage > 3.0 V.

Croconic acid (CA) is an oxocarbon composed of a five‐membered carbon ring with three carbonyl and two enol groups, first isolated as croconic acid dipotassium salt (CA–K_2_) by Gmelin in 1825.^[^ ^18^, ^31^ ^]^ In 2010, S. Horiuchi et al. published a paper in *Nature* stating that CA showed above‐room‐temperature ferroelectricity in a single‐component molecular crystal, which brought significant attention to CA.^[^ ^32^ ^]^ In 2014, Luo et al. demonstrated the first‐time battery application of croconic acid disodium salt (CA–Na_2_), using it as an anode material for sodium‐ion batteries because two carbonyl groups were available as redox centers in the voltage range of 0.7–2.0 V against Na/Na^+^.^[^ ^33^ ^]^ In the same year, Luo et al. demonstrated the two‐electron reactions of CA–Na_2_ with a discharge voltage below 3 V against Li/Li^+^ in *Nano Lett*.^[^ ^34^ ^]^ During cycling, CA–Na_2_ gradually converts to CA dilithium salt (CA–Li_2_) thorough ion exchange. However, while the literature addressed the carbonyl groups of the CA–Li_2_ molecule, it did not investigate the two enolate groups as redox centers, leaving the electrochemical behavior of CA–Li_2_ above 3 V against Li/Li^+^ unexplored. In other words, CA–Li_2_ could have multi‐electron reactions in the high voltage region if the redox reactions of two enolate groups are available above 3 V against Li/Li^+^, resulting in a high theoretical capacity of 638.6 mAh g_CA_^–1^ (**Figure** **1**).

In this study, we investigate the redox reactions of the two enolate groups in CA–Li_2_ by density‐functional theory (DFT) calculations and by cyclic voltammetry (CV) measurements. We show that, with theoretical capacities higher than most known active materials, CA can store four lithium ions and undergo reversible redox reactions near 4 V. We demonstrate the potential of CA as lithium‐ion battery cathode material with a measured discharge voltage plateau of 3.9 V. We report a solvent effect observed to affect CA's storage and discharge capabilities that may be key to future active materials for lithium‐ion batteries.

## 2 Results and Discussion

### 2.1 Redox Potential and Theoretical Energy Density of Croconic Acid by Density Functional Theory Calculations

**Figure 2:**
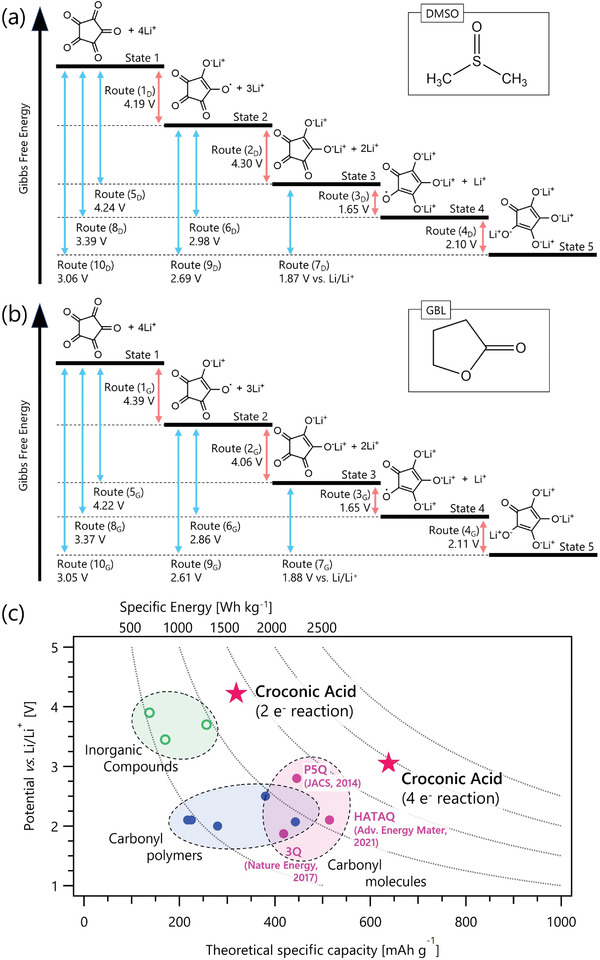
Schematic illustration of the relative heights of the Gibbs free energy and the corresponding redox potential versus Li/Li^+^ of croconic acid for various oxidized states in a) DMSO and b) GBL. Single‐electron (multiple‐electron) reaction routes are indicated with red (blue) arrows. c) The theoretical specific capacities and redox potentials of selected inorganic compounds, carbonyl polymers, and carbonyl molecules including croconic acid. Reproduced with permission (2018, ELSEVIER).^[^ ^40^ ^]^ The red stars show the calculated values for CA in this work. Data are available in Table S2 (Supporting Information).^[^ ^23^, ^24^, ^41^, ^42^, ^43^, ^44^, ^45^, ^46^, ^47^, ^48^, ^49^ ^]^

First, we investigate the redox potentials of the three carbonyls and two enolate groups in the CA–Li_2_ molecule. We calculate the Gibbs free energies for the CA States 1–5 (**Figure** **2**) in two solvents using the gaussian 16 programs with the *ω*B97X‐D function and 6–311G++(d,p) basis set.^[^ ^35^ ^]^ The solvation model based on density is used to calculate CA in dimethyl sulfoxide (DMSO) and *γ*‐butyrolactone (GBL), both of which are stable at the high potential region around 4.5 V against Li/Li^+^.^[^ ^36^, ^37^, ^38^, ^39^ ^]^ The theoretical redox potentials ($E_{x}^{0}$, where *x* = CA or Li) are calculated from the difference of the Gibbs free energies (Δ*_r_G*) before and after the redox reactions using Equation 1 and 2:

(1) $$x+ne^{-}⇌x^{n-},\;\mathrm{and}$$

(2) $$E_{x}^{0}=-\frac{\Delta_{r}G}{nF}$$

The calculated redox potentials of CA are converted against Li/Li^+^ by using Equation 3:

(3) $$E_{\mathrm{CA}}^{0^{\prime }}\left[vs.\;\mathrm{Li}/Li^{+}\right]=E_{\mathrm{CA}}^{0}-E_{\mathrm{Li}}^{0}$$

We test the validity of this approach by calculating the theoretical redox potentials for benzoquinone against Li/Li^+^ in DMSO (Figure S1, Supporting Information), which shows that the errors between the calculated and the experimental redox potentials are only 2.6–3.6% for this molecule.

Routes 1–4 in Figure 2a,b show the calculated potentials of the single‐electron reactions. The redox potentials of the reactions by the two carbonyl groups in CA–Li_2_ (Routes 3 and 4) are lower than 3.0 V, which are consistent with the previous reports.^[^ ^33^, ^34^ ^]^ For both Routes 3_D_ (in DMSO) and 3_G_ (in GBL), $E_{\mathrm{CA}}^{0^{\prime }}\;$= 1.65 V, while for Route 4_D_ and 4_G_, $E_{\mathrm{CA}}^{0^{\prime }}\;$= 2.10 and 2.11 V, respectively. The redox potentials of the single‐electron reactions by the two enolate groups (Routes 1 and 2) are higher than 4.0 V. For Routes 1_D_ and 1_G_, $E_{\mathrm{CA}}^{0^{\prime }}$ = 4.19 and 4.39 V, respectively, while for Routes 2_D_ and 2_G_, $E_{\mathrm{CA}}^{0^{\prime }}$ = 4.30 and 4.06 V, respectively. Routes 5–10 in Figure 2 show the calculated potentials of the multiple‐electron reactions. Unlike the case of one‐electron reactions, the redox potentials of the multi‐electron reactions are similar in both DMSO and GBL.

In DMSO (Figure 2a), Routes 5_D_, 6_D_, and 7_D_ show the two‐electron reactions with $E_{\mathrm{CA}}^{0^{\prime }}$ = 4.24 V, 2.98 V, and 1.87 V, respectively; Routes 8_D_ and 9_D_ show the three‐electron reactions with $E_{\mathrm{CA}}^{0^{\prime }}$ = 3.39 and 2.69 V, respectively; and Route 10_D_ shows the four‐electron reaction with $E_{\mathrm{CA}}^{0^{\prime }}\;$= 3.06 V. In GBL (Figure 2b), Routes 5_G_, 6_G_, and 7_G_ show the two‐electron reactions with $E_{\mathrm{CA}}^{0^{\prime }}$ = 4.22, 2.86, and 1.88 V, respectively; Routes 8_G_ and 9_G_ show the three‐electron reactions with $E_{\mathrm{CA}}^{0^{\prime }}$ = 3.37 and 2.61 V, respectively; and Route 10_G_ shows the four‐electron reaction with $E_{\mathrm{CA}}^{0^{\prime }}$ = 3.05 V.

The above results from the DFT calculations in Figure 2 show all the possible routes for CA redox reactions, which are to be tested experimentally using CV. We note that the two enolate groups in CA–Li_2_ have high theoretical redox potentials above 4.0 V.

Next, we investigate the material‐level theoretical energy densities (*ED*_mater_) of CA in GBL for two scenarios: Scenario 1 considers CA–Li_2_ storing two lithium ions by the two enolate groups, and Scenario 2 considers CA storing four lithium ions by the two enolates and the two carbonyl groups. In Scenario 1, the *ED*_mater_ is calculated to be 1349 Wh kg^–1^, where two one‐electron reactions (Routes 1_G_ and 2_G_) proceed with the average potential of 4.23 V when paired with a lithium‐metal anode. In Scenario 2, the *ED*_mater_ is calculated to be 1949 Wh kg^–1^, where two one‐electron reactions (Routes 1_G_ and 2_G_) proceed with the average potential of 4.23 V, and one two‐electron reaction (Route 7_G_) proceeds at 1.88 V, because the redox potential of Route 4_G_ is higher than that of Route 3_G_, suggesting that the reduction reaction (Route 4_G_: State 4 → State 5) should be followed right after the reduction reaction (Route 3_G_: State 3 → State 4) happens.

We note that the two *ED*_mater_ of the CA molecule calculated above (1349 Wh kg^–1^ and 1949 Wh kg^–1^) are higher than the theoretical *ED*_mater_ of both conventional inorganic intercalation‐based compounds (≈1000 Wh kg^–1^) and recently‐reported high‐energy‐density carbonyl molecules (≈1300 Wh kg^–1^), as shown in Figure 2c.^[^ ^23^, ^24^, ^41^, ^42^, ^43^, ^44^, ^45^, ^46^, ^47^, ^48^, ^49^ ^]^ The theoretical *ED*_mater_ of CA satisfies both Japan's NEDO and the US DOE's Battery500 Consortium target of 500 Wh kg_cell_^–1^ for electrical‐vehicle applications.^[^ ^40^, ^50^, ^51^ ^]^

### 2.2 Determination of Reaction Pathway by Cyclic Voltammetry

**Figure 3:**
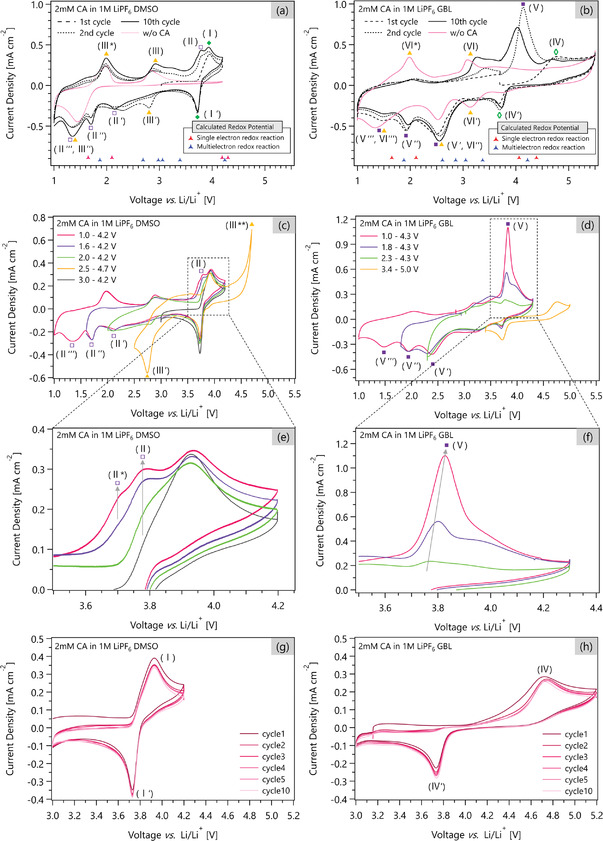
The CV curves of a) 2 × 10^−3^ m CA in 1 m LiPF_6_ DMSO in 1.0–4.2 V versus Li/Li^+^ for the first, second, and tenth cycles, b) 2 × 10^−3^ m CA in 1 m LiPF_6_ GBL in 1.0–5.5 V versus Li/Li^+^ for the first, second, and tenth cycles. The CV curves of the electrolyte without CA are shown as red traces in the corresponding figures. The DFT‐calculated redox potentials are shown as triangles at the bottom of the figures. The corresponding redox peaks are marked with the same symbols. The CV curves measured in the various voltage ranges for c) 2 × 10^−3^ m CA in 1 m LiPF_6_ DMSO, and d) 2 × 10^−3^ m CA in 1 m LiPF_6_ GBL. e,f) show the magnified figures of (c) and (d), respectively. The voltage‐limited CV curves of g) 2 × 10^−3^ m CA in 1 m LiPF_6_ DMSO in 3.0–4.2 V versus Li/Li^+^ for the first ten cycles, and h) 2 × 10^−3^ m CA in 1 m LiPF_6_ GBL in 3.0–5.2 V versus. Li/Li^+^ for the first ten cycles. For each measurement, a platinum disk electrode is used as the working electrode and lithium metal pressed on a Cu mesh works as a counter and reference electrode. The scan rate is 100 mV s^–1^.

First, we investigate the redox reactions of CA in DMSO. The black traces in **Figure** **3**a show the CV curves of 2 × 10^−3^ m CA in 1 m LiPF_6_ DMSO electrolyte in the range of 1.0–4.2 V for the first, second, and tenth cycles. All three curves show four anodic peaks and five cathodic peaks (peak assignments in DMSO are summarized in **Table** **1**). Because the CV curve of the electrolyte without CA (red trace in Figure 3a) also shows Peaks (III), (III*), and (III"), we conclude that these peaks do not originate from CA. Further, when the CV voltage at the high end is raised to 4.8 V, the 1 m LiPF_6_ DMSO electrolyte shows a cathodic Peak (III'), implying that this peak is also not derived from CA (Figure S2, Supporting Information). These peaks could be caused by the chemicals generated by the decomposition of the electrolyte, the inevitable impurities in the electrolyte, or Li salt.

**Table 1:** Cyclic voltammetry peak assignments for CA in DMSO electrolyte

Anodic peak	Anodic peak assignment	Cathodic peak	Cathodic peak assignment
Peak (I)	Route (1)	Peak (I’)	Route (1)
Peak (II) and (II*)	Route (2), (6), (9)	Peak (II’)	Route (2)
Peak (III)	Electrolyte	Peak (II’’)	Route (3)
Peak (III*)	Electrolyte	Peak (II’’’)	Route (4)
‐	‐	Peak (III’)	Electrolyte
‐	‐	Peak (III’’)	Electrolyte

To determine the redox peak pairs of CA, we measure the CV curves at several voltage ranges (Figure 3c,e). Because the CV curve in 3.0–4.2 V (black trace in Figure 3c) shows both the anodic Peak (I) and the cathodic Peak (I'), we conclude that these two peaks are paired.

When the CV voltage at the low end is extended to 2.0, 1.6, and 1.0 V, the height of the anodic Peak (II) is observed to increase accordingly (Figure 3e). In addition, when the CV voltage at the low end is extended to 1.0 V (red trace in Figure 3e), the appearance of the anodic Peak (II*) is observed. These results indicate that the cathodic Peak (III''), which does not originate from CA, overlaps the CA‐originated cathodic Peak (II'''). They also indicate that the cathodic Peaks (II'), (II''), and (II''') are paired with the anodic Peak (II).

During the reduction process, assuming that only one‐electron reactions proceed, we assign the cathodic Peak (I') to Route 1_D_, the cathodic Peak (II') to Route 2_D_, the cathodic Peak (II'') to Route 3_D_, and the cathodic Peak (II''') to Route 4_D_.

**Figure 4:**
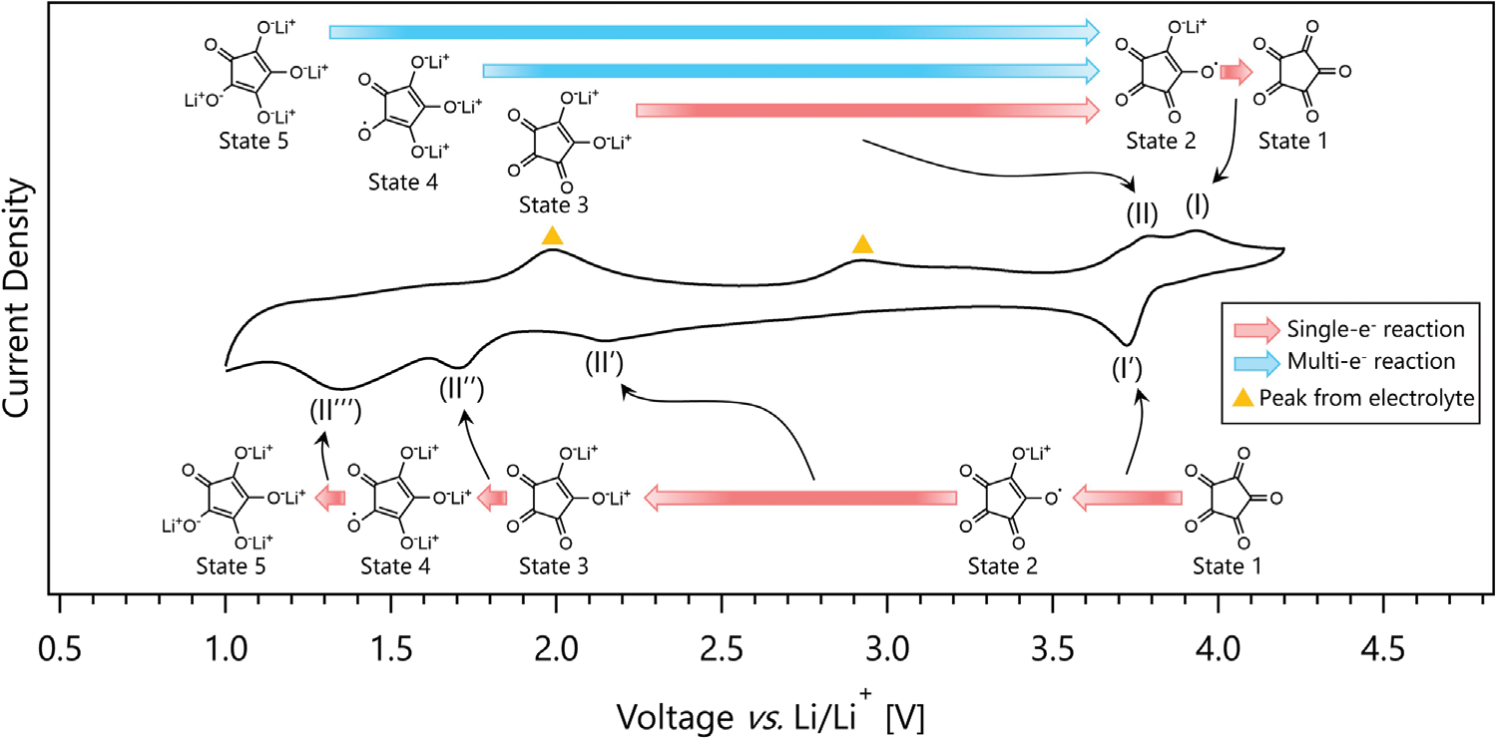
Schematic illustration of the reaction pathways of CA during oxidation and reduction processes in 1 m LiPF_6_ DMSO electrolyte. The reaction pathways are the same for both DMSO and 1 m LiPF_6_ GBL electrolytes.

During the oxidation process, the pairing of the cathodic Peaks (II'), (II''), and (II''') with the anodic Peak (II) suggests that Peak (II) derives from Routes 2_D_, 6_D_, and 9_D_; Peak (II) is from Route 2_D_ when CA is reduced to State 3, from Route 6_D_ when reduced to State 4, and from 9_D_ when reduced to State 5. The pairing of the anodic Peak (I) with the cathodic Peak (I') suggests that it originates from Route 1_D_. **Figure** **4** illustrates the redox pathways of CA in DMSO determined by the CV measurements.

What makes the reduction potential of Route 2_D_ obtained by CV different from the calculated value could be the presence of stable intermediates. Our calculations are based only on thermodynamics, and the redox potentials are calculated from the difference in Gibbs free energy between the initial and final structures. However, when a stable intermediate exists and kinetics is taken into account, the redox potential will be determined by the difference in Gibbs free energy before and after the rate‐limiting reaction involving the stable intermediate.

To evaluate the reversibility of the redox reaction around 4 V, we repeat the CV in the 3.0–4.2 V range for ten cycles (Figure 2g). At the tenth cycle, the current density of the anodic Peak (I) is 95.2% of that at the second cycle, while the current density of the cathodic Peak (I') is 105.4% of that at the second cycle. These results suggest that the Route 1_D_ redox reaction is reversible near 4 V and both States 1 and 2 of CA are stable.

Next, we investigate the redox reactions of CA in GBL. The black traces in Figure 3b show the CV curves of 2 × 10^−3^ m CA in 1 m LiPF_6_ GBL electrolyte in the range of 1.0–5.5 V for the first, second, and tenth cycles. All three curves show four anodic peaks and five cathodic peaks (peak assignments in GBL are summarized in **Table** **2**). Because the CV curve of the electrolyte without CA (red trace in Figure 3b) also shows Peaks (VI), (VI*), (VI'), (VI''), and (VI'''), we conclude that these peaks do not originate from CA.

**Table 2:** Cyclic voltammetry peak assignments for CA in GBL electrolyte

Anodic peak	Anodic peak assignment	Cathodic peak	Cathodic peak assignment
Peak (IV)	Route (1)	Peak (IV’)	Route (1)
Peak (V)	Route (2), (6), (9)	Peak (V’)	Route (2)
Peak (VI)	Electrolyte	Peak (V’’)	Route (3)
Peak (VI*)	Electrolyte	Peak (V’’’)	Route (4)
‐	‐	Peak (VI’)	Electrolyte
‐	‐	Peak (VI’’)	Electrolyte
‐	‐	Peak (VI’’’)	Electrolyte

To determine the redox peak pairs of CA, we measure the CV curves at several voltage ranges (Figure 3d,f). Because the anodic Peak (V) disappears while the cathodic Peak (IV') and the anodic Peak (IV) remain during the CV measurement in a range of 3.4–5.0 V (yellow trace in Figure 3d), we conclude that the cathodic Peak (IV') and the anodic Peak (IV) are paired. When the CV voltage at the low end is extended to 2.3 and 1.8 V, the height of the anodic Peak (V) is observed to increase accordingly and shift toward the high end (Figure 3f). These results indicate that the cathodic Peaks (VI') and (VI'''), which do not originate from CA, overlap with the CA‐originated cathodic Peaks (V') and (V'''). They also indicate that the cathodic Peaks (V'), (V''), and (V''') are paired with the anodic Peak (V). Because the relative CV peak positions of CA do not change for both DMSO and GBL, Figure 4 also illustrates the redox pathways of CA in GBL determined by the CV measurements.

To evaluate the reversibility of the redox reaction around 4.2 V, we repeat the CV in the 3.0–5.2 V range for ten cycles (Figure 3h). At the tenth cycle, the current density of the anodic Peak (IV) at the tenth cycle is 94.5% of that at the second cycle, while the current density of the cathodic Peak (IV') is 113.2% of that at the second cycle. This suggests that the Route 1_G_ redox reaction is reversible near 4 V and both States 1 and 2 of CA are stable.

Although CA follows the same reaction pathways in both electrolytes, the relative heights of the anodic Peak (V) are considerably higher than Peak (II). By calculating the ratios of the peak areas, we can quantitatively evaluate how many electrons and Li ions are withdrawn during oxidation. In DMSO, the ratio of the total area of Peaks (II) and (II*) to the total areal of Peaks (II'), (II''), and (II''') is 0.39, suggesting that at least 39% of the electrons and lithium ions, stored through the reduction process (Routes 2_D_, 3_D_, and 4_D_), are withdrawn through the oxidation process (Routes 2_D_, 6_D_, and 9_D_). We note that the actual value must be higher than the calculated ratio because of the overlap of the cathodic peaks with peaks from the electrolyte. In GBL, the ratio of the area of Peak (V) to the total areal of Peaks (V'), (V''), and (V''') is 0.58, suggesting that at least 58% of the electrons and lithium ions are withdrawn through the same processes as in DMSO. From these results, we conclude that electrons and lithium ions stored by CA are more readily withdrawn in GBL than in DMSO, possibly due to the formation of an interface between CA and GBL solvent molecules that conducts both electrons and lithium ions. We note that further investigation on this interface between CA and solvent molecules is required to understand why GBL is a solvent that promotes electron and lithium‐ion storage compared to DMSO.

Next, we summarize our findings from both DFT calculations and CV measurements. While CA is reduced by one‐electron reactions exclusively, CA is oxidized to State 2 in one step, and then to State 1 by a one‐electron reaction. For example, when CA is reduced to State 5, the oxidation pathway is State 5 → State 2 → State 1. When CA is reduced to State 4, the oxidation pathway is State 4 → State 2 → State 1. When CA is reduced to State 3, the oxidation pathway is State 3 → State 2 → State 1 (Figure 4). These observations combined with our reversibility tests suggest that both States 1 and 2 of CA are stable. The stabilities of States 1 and 2 at voltages near 4 V are desirable when considering the battery application of CA. Our finding that the lithium ions stored by CA are more readily withdrawn in GBL than in DMSO suggests that the formation of an electrical and Li‐ion conductive interface between CA and solvent is essential.

### 2.3 Croconic Acid as Cathode Material for High‐Voltage Lithium‐Ion Batteries

**Figure 5:**
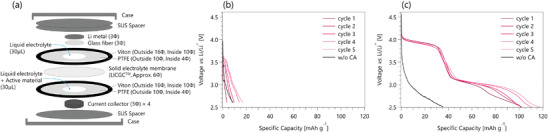
a) Schematic illustration of the two‐compartment cell. The red traces in (b) and (c) show the discharge curves at 20 mA g^–1^ for 10 × 10^−3^ m CA in 1 m LiPF_6_ DMSO and 10 × 10^−3^ m CA in 1 m LiPF_6_ GBL, respectively. The discharge curves of the electrolyte without CA are shown as black traces in the same figure.

We interrogate the battery performance of CA as a cathode material for lithium‐ion batteries using a two‐compartment cell designed for measuring organic small molecules.^[^ ^52^ ^]^ As **Figure** **5**a shows, the positive compartment is filled by 10 × 10^−3^ m CA in 1 m LiPF_6_ DMSO (or GBL), while the negative compartment is filled by 1 m LiPF_6_ DMSO (or GBL). The two compartments are separated by a solid electrolyte membrane made of lithium‐ion conductive glass‐ceramics (LICGC^TM^, OHARA) to prevent CA from reaching the lithium metal counter electrode. A carbon‐coated Al foil in a four‐layer configuration is used as the current collector to minimize the diffusion length of the dissolved active material.^[^ ^52^ ^]^ The battery performances are measured in the voltage range of 2.6–4.0 V in DMSO and the voltage range of 2.5–4.7 V in GBL. The cell is charged by keeping it at a constant voltage until an amount of electricity equivalent to a one‐electron reaction (188.6 mAh g_CA_^–1^) flows.

In DMSO, the discharge curves are observed as near straight lines with a negative slope (red traces in Figure 5b). The discharged curves resemble the curve acquired without CA (black trace in Figure 5b). The discharge capacity is measured to be 3.4 mAh g_CA_^–1^ for the first cycle and 17.0 mAh g_CA_^–1^ for the fifth cycle, indicating that CA does not store energy efficiently in DMSO. In GBL, the discharge curves are observed as stepped lines descending from left to right with two plateaus at 3.9 and 3.1 V (red traces in Figure 5c). Compared with the discharge curve of the electrolyte without CA (black trace in Figure 5c), we conclude that these two plateaus originate from the CA reduction reactions. Please note that carbon‐coated Al foils are used for the battery galvanostatic discharge tests, while a Pt disk electrode is used for CV tests, which could result in different working potentials by changing the electrochemical behavior of CA. The discharge capacity is measured to be 102.2 mAh g_CA_^–1^ for the first cycle and 117.6 mAh g_CA_^–1^ for the fifth cycle. The plateau at 3.9 V shows cyclability with no capacity loss even after five cycles. The plateau capacity at 3.1 V gradually increases with each cycle, which is probably because more electrons are consumed to oxide CA during the charging process while the side oxidation reactions during the charging process subside over the cycles. The potential side reactions during the constant‐voltage charging process could be the oxidation reactions of the electrolyte, amorphous carbon on current collectors, and stainless steel coin cell.

These results indicate that the superior battery performance of CA in GBL over DMSO is related to the higher propensity of CA for electron and lithium‐ion storage in GBL, supporting the conclusions drawn from our CV measurements above. They also suggest that understanding the interface between CA and solvent molecules may hold the key information toward designing future high‐performance organic active materials.

**Table** **3** compares the discharge voltages of CA measured here with other state‐of‐the‐art organic small molecules that undergo multi‐electron redox reactions. While most of the organic small molecules reported elsewhere have discharge voltages below 3.0 V against Li/Li^+^, CA exhibits considerably higher average discharge voltages of 3.9 and 3.24 V for one‐ and two‐electron reactions, respectively, indicating that CA has the potential to function as a high‐voltage cathode material for a high‐energy‐density battery.

**Table 3:** The average discharge voltages of croconic acid along with the state‐of‐the‐art organic molecules whose theoretical capacities are higher than 400 mAh g^–1^

Molecules	Average discharge voltage [V vs. Li/Li^+^]	Reference
3Q	1.88	*Nature Energy* (2017)^[^ ^23^ ^]^
HATAQ	2.10	*Adv. Energy Mater*. (2021)^[^ ^24^ ^]^
DB‐1	2.60	*J. Power Sources* (2021)^[^ ^30^ ^]^
P5Q	2.60	*JACS* (2014)^[^ ^49^ ^]^
**CA (1 electron)**	**3.90**	**This work**
**CA (2 electron)**	**3.24**	**This work**

## 3 Conclusions

DFT calculations and CV measurements in DMSO and GBL‐based electrolytes reveal that croconic acid (CA) has a potential as the 4 V‐class organic cathode material using the redox reactions of the two enolate groups. The battery‐performance tests show that in the GBL‐based electrolyte, CA undergoes repeatable discharge plateau at 3.9 and 3.1 V with the discharge capacity of ≈100 mAh g_CA_^–1^. Since no organic active material is known to undergo redox reactions in the high voltage range around 4 V, CA has a great potential to function as a cathode material for high‐energy‐density batteries, satisfying the world's target of 500 Wh kg_cell_^–1^ for electrical‐vehicle applications. Further research should be conducted to use CA effectively as a low‐cost, sustainable, and environmentally friendly energy storage material.

## Conflict of Interest

The authors declare no conflict of interest.

## Author Contributions

Y.K. was associated with investigations and wrote the original draft. H.K. was associated with methodology and reviewed and edited the final manuscript. K.I. and Y.G. reviewed and edited the final manuscript. I.H. was associated with conceptualization, funding, supervision, and reviewed and edited the final manuscript.

## Supporting information

Supporting Information

## Data Availability

The data that support the findings of this study are available from the corresponding author upon reasonable request.
