# Supplementary material for: Are Redox‐Active Organic Small Molecules Applicable for High‐Voltage (>4 V) Lithium‐Ion Battery Cathodes?
Source: Adv Sci (Weinh). 2022 Mar 10;9(12):2200187. doi: 10.1002/advs.202200187 (PMC9036039; doi:10.1002/advs.202200187)
Supplement: Supplementary file 1 — Supporting Information [file ADVS-9-2200187-s001.pdf]

ADVANCED SCIENCE
Open Access

Supporting Information

for *Adv. Sci.*, DOI 10.1002/advs.202200187

Are Redox-Active Organic Small Molecules Applicable for High-Voltage (>4 V) Lithium-Ion Battery Cathodes?

Yuto Katsuyama, Hiroaki Kobayashi, Kazuyuki Iwase, Yoshiyuki Gambe and Itaru Honma**

Supporting Information

Are Redox-Active Organic Small Molecules Applicable for High-Voltage (>4 V) Lithium-ion Battery Cathodes?

Yuto Katsuyama, Hiroaki Kobayashi, Kazuyuki Iwase, Yoshiyuki Gambe, and Itaru Honma**

S1. Experiments

S1.1 DFT calculations

The redox potentials of the three carbonyl and two enolate groups in croconic acid dilithium salt (CA–Li_2) are investigated by DFT calculations. The Gibbs free energies for States 1–5 in **Figure 1** in the main manuscript are calculated on the software (gaussian 16) using ωB97X-D function and 6–311G++(d,p) basis set. The SMD solvation model is used to calculate the Gibbs free energies in dimethyl sulfoxide (DMSO) and γ–butyrolactone (GBL) solvents. Gaussian 16 offers several pre-defined solvents including DMSO. However, because GBL is not included in the list, it is necessary to specify the solvent by adding the following seven parameters.

Additional Input for SMD solvation model (GBL)

Eps=41.70

EpsInf=2.0566

HbondAcidity=0.00

HbondBasicity=0.54

SurfaceTensionAtInterface=64.19367

CarbonAromaticity=0.00

ElectronegativeHalogenicity=0.00

Table S1. Symbol definition[1] and references

Symbols	Definition	Reference
Eps	Dielectric constant at 298 K	[2]
EpsInf	The square of the index of refraction at optical frequencies (293 K)	[3,4]
HbondAcidity	Abraham's hydrogen bond acidity	[5]
HbondBasicity	Abraham's hydrogen bond basicity	[5]
SurfaceTensionAtInterface	$\gamma = \gamma_m/\gamma_o$<br>$\gamma_m$: macroscopic surface tension at a liquid-air interface at 298 K<br>γ_o: 1 cal mol^{-1}Å^{-2}	[6]
CarbonAromaticity	Aromaticity	-
ElectronegativeHalogenicity	Electronegative halogenicity	-

Aromaticity is defined as a fraction of non-hydrogenic solvent atoms that are aromatic carbon atoms.[1] Therefore, the value for aromaticity of GBL is 0/7. Electronegative halogenicity is defined as a fraction of non-hydrogenic solvent atoms that are F, Cl, or Br.[1] Hence, the value is 0/7.

The theoretical redox potentials are calculated from the difference of the Gibbs free energies (ΔG) before and after the redox reactions using equation (2) in the main manuscript. The obtained redox potentials are subtracted by the redox potential of Li/Li^+ in the same solvent to get the redox potential against Li/Li^+. ωB97X-D function and 6–31G++(d,p) basis set are employed to calculate the Gibbs free energy of Li metal and Li ion in the solvents.

S1.2 Calculation of theoretical energy density

The specific capacities and redox potentials are shown in the **Table S2**. They are calculated when paired with a lithium-metal anode whose specific capacity is 3860 mA h g^{-1} at 0.0 V *vs.* Li/Li^{+}.

Table S2. List of materials plotted on Figure 2(c)

Materials	Theoretical specific capacity [mAh g^{-1}]	Average working voltage [V]	Theoretical energy density [Wh kg^{-1}]	Reference
LCO (0.5 e– reaction)	137	3.9	534.3	[7]
NMC (1 e– reaction)	256.5	3.7	949.0	[8]
LFP	170	3.45	586.5	[9]
PVBDT	217	2.1	455.7	[10]
PI	280	2.0	560.0	[11]
PI-2	443	2.07	916.9	[12]
PAQS	225	2.1	472.5	[13]
ATQS	380	2.5	949.9	[14]
3Q	418.2	1.87	781.9	[15]
P5Q	446	2.8	1248.7	[16]
HATAQ	514.3	2.1	1079.9	[17]

S1.3 Cyclic voltammetry tests

All experiments are carried out in an Ar-filled glove box. CA is purchased from Tokyo Chemical Industry and used without further purification. $LiPF_6$ salt, DMSO and GBL are purchased from Kishida Chemical Co., Ltd, FUJIFILM Wako Chemical Corp., and Kanto Chemical Co., Inc., respectively. Two types of electrolytes (2 mM CA in 1 M $LiPF_6$ DMSO and 2 mM CA in 1 M $LiPF_6$ GBL) are prepared. A platinum disk electrode with a 3 mm diameter (BAS Inc.) is used for a working electrode, and a polished lithium metal pressed on a copper mesh is used for a counter and reference electrode. Electrolyte (2 mL), platinum disk electrode, and lithium electrode are set in a 5 mL screw vial bottle. CV tests are carried out by using a potentiostat (VMP3, Bio-Logic) with a scan rate of 100 mV sec^{-1}.

S1.4 Two-compartment half-cell tests

Two-compartment cells, whose configuration is shown in **Figure 3(a)** in the main manuscript, are fabricated in an Ar-filled glove box. The positive compartment is filled with 30 μL of 10 mM CA in 1 M $LiPF_6$ DMSO or 10 mM CA in 1 M $LiPF_6$ GBL, and the negative compartment is filled with 30 μL of electrolytes without active material (1 M $LiPF_6$ DMSO or 1 M $LiPF_6$ GBL). Positive and negative compartments are separated by a lithium-ion conductive glass ceramics (LICGC™, OHARA), so that CA does not shuttle by reaching lithium metal. In the positive compartment, carbon-coated Al foils are used for current collectors. By layering four current collectors, the diffusion length of the dissolved active material is shortened.[18] In the negative compartment, lithium metal with a 3 mm diameter is pressed on a SUS spacer, and a glass fiber (GA55, Advantec), with a 3 mm diameter is placed between lithium metal and LICGC. Fabricated cells are tested by using a potentiostat/galvanostat (VMP3, Bio-Logic). The cell is charged by keeping at a constant voltage until an amount of electricity equivalent to a one-electron reaction (188.6 mAh g_{CA}^{-1}) flows. The positive electrode is charged at constant voltage of 4.0 V or 4.7 V against Li/Li^+ for DMSO or GBL-based electrolytes, respectively. After charging, the cell is discharged with a constant current density of 20 mA g_{CA}^{-1}. To clarify the contribution of CA, electrolytes without CA are charged and discharged in the same way. The amount of electricity flowing during charging and the constant current density during discharging are determined assuming that the electrolyte contained 10 mM of CA. Therefore, it can be considered that all the electricity flown is consumed by the side reactions, which is not originated from CA. The discharge profiles of electrolytes not containing CA are drawn in **Figure 3 (b–c)** by black lines.

S2. Validity of the DFT calculation results

The validity of the calculation results is evaluated using benzoquinone. The calculated redox potential and the Gibbs free energies in DMSO solvent are illustrated in Figure S1 (a). The calculation results show that the redox potentials of the single-electron reactions by the two carbonyl groups in benzoquinone (State 1 ⇄ State 2, State 2 ⇄ State 3) are 3.07 V and 2.73 V against Li/Li^+, respectively. The CV curve of 2 mM benzoquinone in 1 M $LiPF_6$ DMSO solvent is shown in Figure S1 (b). The obtained redox potentials are 2.96 V and 2.66 V *vs*. Li/Li^+. The errors between the calculated and the experimental redox potentials are 3.6% and 2.6%, respectively.

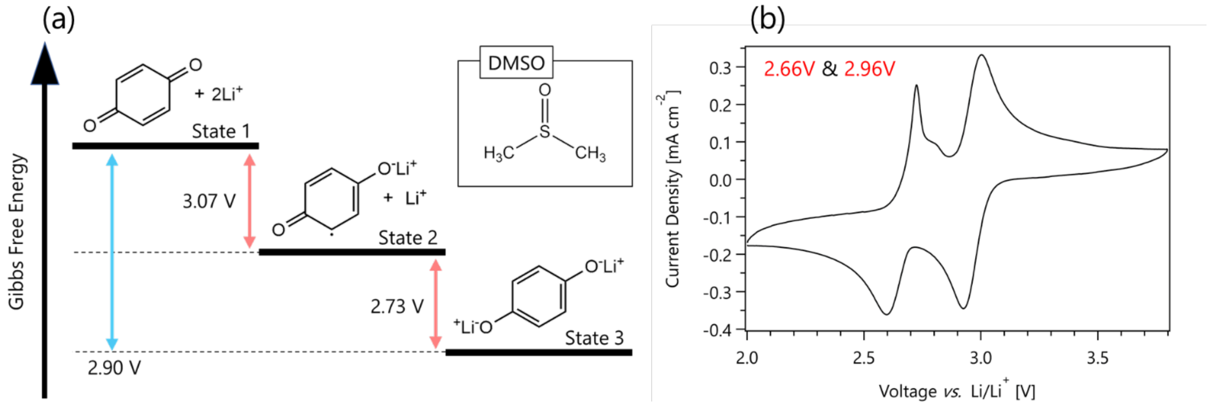

Figure S1. (a) Schematic illustration of the relative height of the Gibbs free energy and the corresponding redox potential *vs*. Li/Li^+ of benzoquinone for various oxidized states in DMSO solvent. Single-electron reactions are expressed with red arrows, and multi-electron reactions are described with blue arrows. (b) Cyclic voltammetry curve of 2 mM benzoquinone in 1 M $LiPF_6$ DMSO in 2–3.8 V *vs.* Li/Li^+ for the 2nd cycle. Platinum disk electrode is used as a working electrode, and lithium metal pressed on Cu mesh works as a counter and reference electrode. The scan rate is 100 mV sec^{-1}.

S3. Various-voltage-range CV curves of 1 M $LiPF_6$ DMSO solvent

The CV curves of 1 M $LiPF_6$ DMSO in the range of 1.0–4.8 V and 1.0–4.2 V against Li/Li^+ are displayed in **Figure S2**. The CV curve in 1.0–4.2 V *vs*. Li/Li^+ does not show the cathodic peak (III'). However, it appears when a CV is tested in 1.0–4.8 V vs. Li/Li^+.

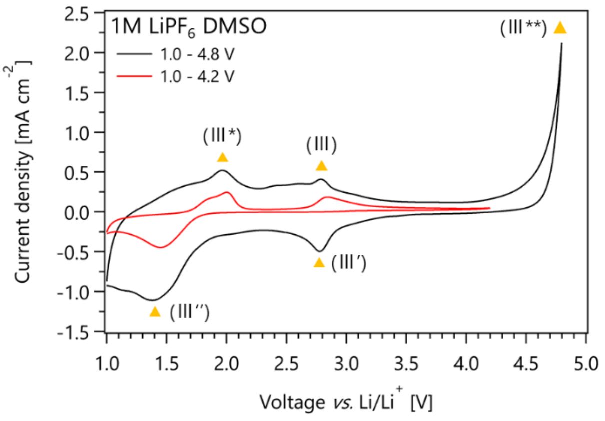

Figure S2. CV curves of 1 M $LiPF_6$ DMSO in the range of 1.0–4.8 V (black line) and 1.0–4.2 V against Li/Li^+ (red line). The scan rate is 100 mV sec^{-1}.

S4. Calculation of ratio of peak areas

In order to quantitatively evaluate how many electrons and Li ions are withdrawn from CA during oxidation through State 2 ⇌ State 5, the ratio of the peak areas is calculated. All curves are fitted by Cauchy distribution.

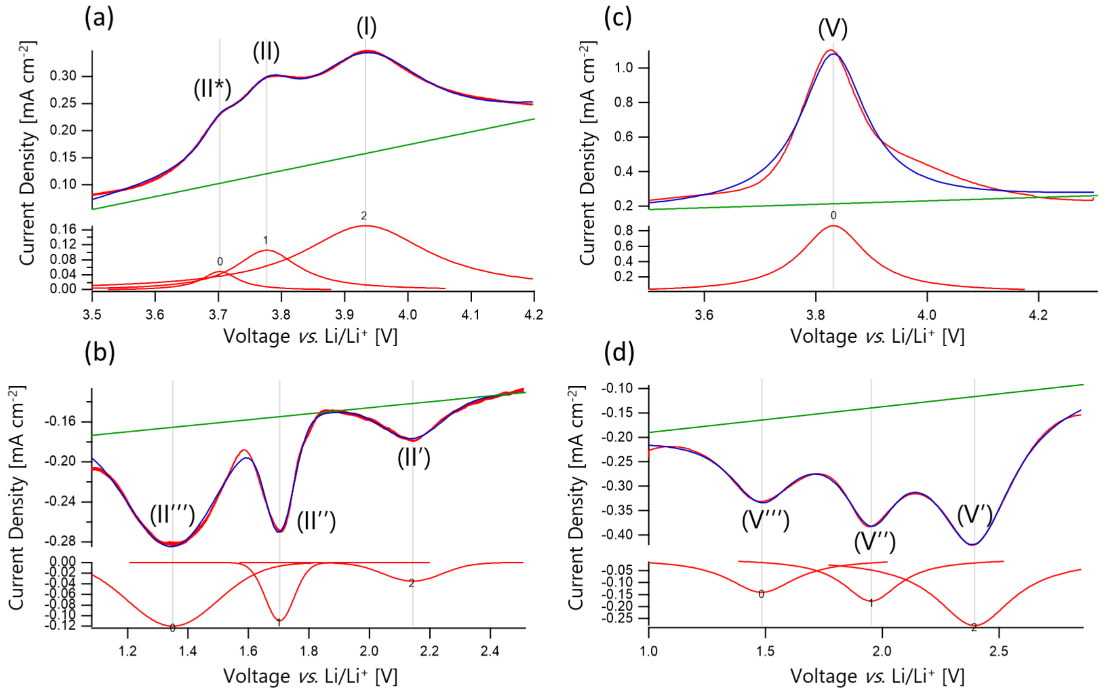

Figure S3. Peak separation and fitting by Cauchy distribution of (a) peak (I) and (II), (b) peak (II'), (II'') and (II'''), (c) peak (V) and (d) peak (V'), (V'') and (V''').

<u>DMSO</u>

Figure S3 (a) is the anodic CV curve in the range of 3.5–4.2 V. The curve is fitted by assuming that there are three anodic peaks (I), (II) and (II*). Figure S3 (b) is the cathodic CV curve in the range of 1.1–2.5 V. The curve is fitted by assuming that there are three cathodic peaks (II'), (II'') and (II'''). The fitted peak areas are shown in Table S3. From the obtained numbers, the ratio of the peak areas can be calculated as follows;

$$\text{Peak area ratio (DMSO)} = \frac{\text{Total area of anodic peak (II) and (II} *)}{\text{Total areal of cathodic peak (II}'), (\text{II}'')\text{and (II}''')}$$

$$= \frac{0.02017 + 0.00536}{0.04403 + 0.01298 + 0.00843} = 0.39001 \approx 0.39$$

Table S3. Fitted peak areas in DMSO solvents.

	Peak	Peak area
Anodic	Peak (I)	0.06500
	Peak (II)	0.02017
	Peak (II*)	0.00536
Cathodic	Peak (II')	0.00843
	Peak (II'')	0.01298
	Peak (II''')	0.04403

GBL

Figure S3 (c) is the anodic CV curve in the range of 3.5–4.3 V. The curve is fitted by assuming that there is an anodic peak (V). Figure S3 (d) is the cathodic CV curve in the range of 1.1–2.5 V. The curve is fitted by assuming that there are three cathodic peaks (V', (V'') and (V'''). The fitted peak areas are shown in Table S4. From the obtained numbers, the ratio of the peak areas can be calculated as follows;

$$\text{Peak area ratio (GBL)} = \frac{\text{Total area of anodic peak (V)}}{\text{Total areal of cathodic peak (V}'\text{), (V}''\text{)and (V}'''\text{)}}$$

$$= \frac{0.19494}{0.07787 + 0.08163 + 0.17547} = 0.58197 \approx 0.58$$

Table S4. Fitted peak areas in GBL solvents.

	Peak	Peak area
Anodic	Peak (V)	0.19494
Cathodic	Peak (V')	0.07787
	Peak (V'')	0.08163
	Peak (V''')	0.17547

S5. Electrochemical performance of CA when being impregnated in porous carbon

Organic active materials have showed excellent battery performances when being impregnated in porous carbon and being mixed with conductive additives.[19,20] Therefore, we also tried to use CA by impregnating CA in porous carbon. All procedures are conducted in an Ar-filled glove box. First, CA (30mg) is dissolved in acetone (50 mL) and sonicated for 1 hour. Then, activated carbon (MAXSORB, 70 mg) is added into the solvent and dispersed by sonication for 1 hour. The solvent is placed on a hot plate at 69 °C with stirring at 300 rpm so that acetone is evaporated, and CA is impregnated into the pores of the activated carbon. The obtained powder (50 mg) is mixed with 20 mg of Lithium bis(trifluoromethanesulfonyl)imide (LiTFSI) and 20 mg of acetylene black in a motor for 10 minutes. Then, 10 mg of Polytetrafluoroethylene (PTFE) is added and mixed in a motor for another 10 minutes. The obtained pellet is hollowed out using a puncher 7 mm in diameter and pressed on an aluminium mesh current collector. Quasi-solid electrolyte containing ionic liquid is used for a separator. Fumed SiO_2 (7 nm particle size) is mixed with 1 M LiTFSI in EMI-TFSI at the weight ratio of 85:15 in a motor for 15 minutes. It is then mixed with PTFE at the weight ratio of 95:5 for another 15 minutes. The obtained pellet is extended thinly (~200 μm thickness) and is hollowed out by a puncher 10 mm in diameter. 12 Ø lithium metal is used for counter and reference electrode.

The CV curves of the electrodes are shown in Figure S4. Unlike the CV curves in Figure 3 in the main manuscript, there are only two peaks with large polarization.

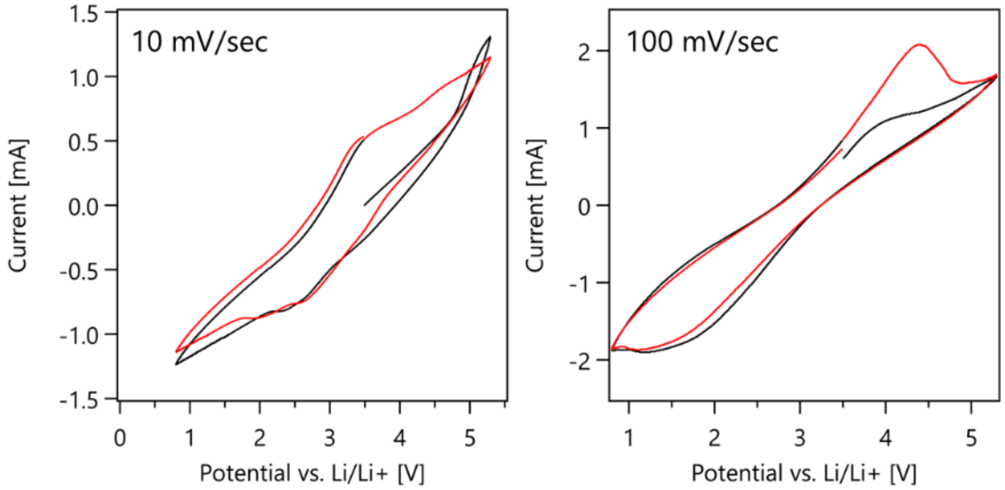

Figure S4. CV curves of the CA–impregnated activated carbon electrodes at 10 mV sec^{-1} (left) and 100 mV sec^{-1} (right). The weight ratio of the electrode is as follows; CA: activated carbon: LiTFSI: acetylene black: PTFE = 15: 35: 20: 20: 10. Black line represents the CV curve at the 1st cycle and the red curve represents that at the 2nd cycle.

S6. Current density transition during the constant-voltage charging

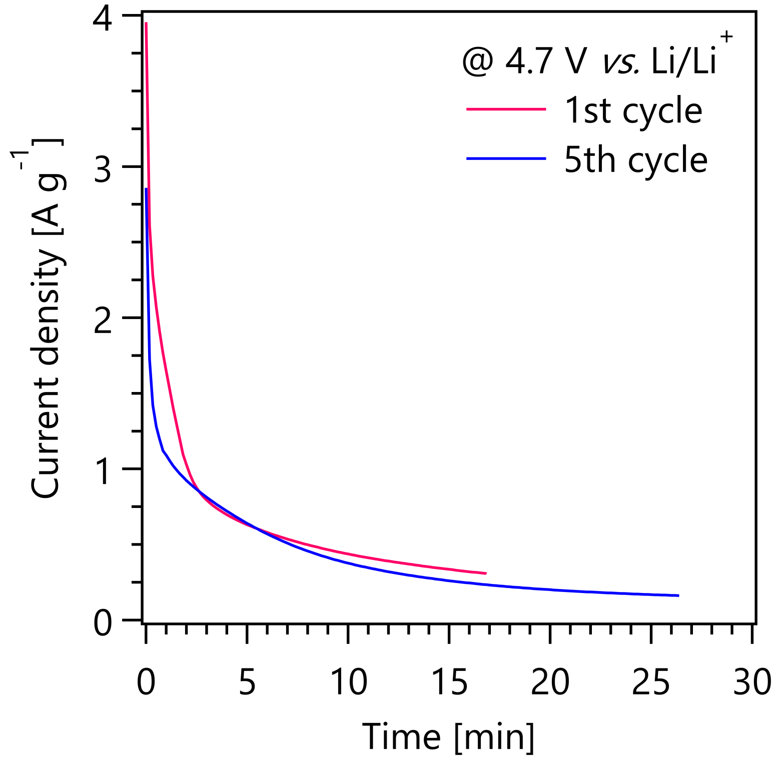

Figure S5. Current density transition during the constant-voltage charging at the 1st and 5th cycles of 2 mM CA in 1.0 M $LiPF_6$ GBL.

References

[1] "Gaussian Tip: Defining Solvents for SMD Calculations | Gaussian.com," can be found under https://gaussian.com/smdtip/.
[2] Ch. Wohlfarth, in *Supplement to IV/6* (Ed.: M.D. Lechner), Springer Berlin Heidelberg, Berlin, Heidelberg, **2008**, pp. 222–223.
[3] W. M. Haynes, D. R. Lide, Eds. , *CRC Handbook of Chemistry and Physics: A Ready-Reference Book of Chemical and Physical Data*, CRC Press, Boca Raton, Fla., **2015**.
[4] S.-K. Fan, H.-P. Lee, C.-C. Chien, Y.-W. Lu, Y. Chiu, F.-Y. Lin, *Lab on a Chip* **2016**, *16*, 847–854.
[5] A. Duereh, Y. Sato, R. L. Smith, H. Inomata, *J. Phys. Chem. B* **2016**, *120*, 4467–4481.
[6] Wiley-VCH Verlag GmbH & Co. KGaA, Ed. , *Ullmann's Encyclopedia of Industrial Chemistry*, Wiley, **2000**.
[7] N. Nitta, F. Wu, J. T. Lee, G. Yushin, *Mater. Today* **2015**, *18*, 252–264.
[8] J. Xu, F. Lin, M. M. Doeff, W. Tong, *J. Mater. Chem. A* **2017**, *5*, 874–901.
[9] B. Lung-Hao Hu, F.-Y. Wu, C.-T. Lin, A. N. Khlobystov, L.-J. Li, *Nat. Commun.* **2013**, *4*, 1687.
[10] B. Häupler, T. Hagemann, C. Friebe, A. Wild, U. S. Schubert, *ACS Appl. Mater. Interfaces* **2015**, *7*, 3473–3479.
[11] N. Liu, Y. Liu, Y. Zhao, Y. Liu, Q. Lan, J. Qin, Z. Song, H. Zhan, *ACS Appl. Mater. Interfaces* **2019**, *11*, 46726–46734.
[12] H. Wu, S. A. Shevlin, Q. Meng, W. Guo, Y. Meng, K. Lu, Z. Wei, Z. Guo, *Adv. Mater.* **2014**, *26*, 3338–3343.
[13] J. Xie, W. Chen, Z. Wang, K. C. W. Jie, M. Liu, Q. Zhang, *Chemistry – An Asian Journal* **2017**, *12*, 868–876.
[14] A. Petronico, K. L. Bassett, B. G. Nicolau, A. A. Gewirth, R. G. Nuzzo, *Adv. Energy Mater.* **2018**, *8*, 1700960.
[15] C. Peng, G.-H. Ning, J. Su, G. Zhong, W. Tang, B. Tian, C. Su, D. Yu, L. Zu, J. Yang, M.-F. Ng, Y.-S. Hu, Y. Yang, M. Armand, K. P. Loh, *Nat. Energy* **2017**, *2*, 17074.
[16] Z. Zhu, M. Hong, D. Guo, J. Shi, Z. Tao, J. Chen, *J. Am. Chem. Soc.* **2014**, *136*, 16461–16464.
[17] M. Wu, N. T. H. Luu, T. Chen, H. Lyu, T. Huang, S. Dai, X. Sun, A. S. Ivanov, J. Lee, I. Popovs, W. Kaveevivitchai, *Adv. Energy Mater.* **2021**, 2100330.
[18] H. Senoh, M. Yao, H. Sakaebe, K. Yasuda, Z. Siroma, *Electrochim. Acta* **2011**, *56*, 10145–10150.
[19] T. Tomai, S. Mitani, D. Komatsu, Y. Kawaguchi, I. Honma, *Sci. Rep.* **2014**, *4*, 3591.
[20] Y. Katsuyama, Y. Nakayasu, K. Oizumi, Y. Fujihara, H. Kobayashi, I. Honma, *Adv. Sustainable Syst.* **2019**, *3*, 1900083.
